# Sarcopenia and Frailty in COPD: Mechanisms, Relationship with Malnutrition and Potential Therapeutic Interventions

**DOI:** 10.3390/nu18122003

**Published:** 2026-06-20

**Authors:** Saoussen Naas, Mónika Fekete, Riad Bejta, Regina Bakos, Borbála Szalai, János Tamás Varga

**Affiliations:** 1 Department of Pulmonology, Semmelweis University, 1085 Budapest, Hungary; naassaoussen@gmail.com (S.N.); riadbejta@gmail.com (R.B.); bakos.regina@semmelweis.hu (R.B.); szalai.bori98@gmail.com (B.S.); 2 Institute of Preventive Medicine and Public Health, Semmelweis University, 1089 Budapest, Hungary; fekete.monika@semmelweis.hu

**Keywords:** COPD, sarcopenia, frailty, malnutrition, protein intake, nutritional supplementation, vitamin D, omega-3 fatty acids, inflammation, nutritional biomarkers

## Abstract

**Background:** Sarcopenia and frailty are highly prevalent extrapulmonary manifestations of chronic obstructive pulmonary disease (COPD) and are strongly associated with reduced exercise tolerance, exacerbation risk, hospitalizations, and mortality. Beyond inflammation, oxidative stress, and physical inactivity, emerging evidence highlights nutrition as a major modifiable driver of muscle deterioration in COPD. Nutritional deficits impair anabolic signaling, exacerbate proteolysis, worsen mitochondrial dysfunction, and contribute to frailty progression. **Methods:** This narrative review synthesizes evidence from PubMed, Embase, Scopus, and Web of Science up to 2025, integrating mechanistic, metabolic, nutritional, and biomarker-related pathways underlying muscle dysfunction in COPD. Studies examining inflammation, hypoxemia, oxidative stress, hormonal imbalance, nutrition, and emerging biomarkers were included. **Results:** COPD-related sarcopenia results from converging inflammatory (TNF-α, IL-6), catabolic (FOXO, UPS), metabolic, and vascular mechanisms, compounded by energy deficiency, protein insufficiency, and micronutrient deficits. Inadequate intake of protein, vitamin D, antioxidants, and omega-3 fatty acids increase anabolic resistance, enhance muscle catabolism, and worsen frailty. Nutritional interventions, particularly high-protein supplementation, leucine-enriched formulas, vitamin D repletion, omega-3 fatty acids, and multimodal nutrition–exercise programs, demonstrate benefits in muscle mass, strength, and physical performance. Biomarkers such as GDF-15, CAF22, and specific microRNAs reflect nutritional status and correlate with muscle health in COPD. **Conclusions:** Sarcopenia and frailty in COPD arise from a complex interplay of inflammatory, metabolic, nutritional, and lifestyle-related factors. Integrating nutritional assessment and targeted dietary interventions with exercise and pulmonary rehabilitation is essential to counteract anabolic resistance and improve functional outcomes. Advances in biomarker research may support earlier diagnosis and personalized nutrition-based therapeutic strategies.

## 1. Introduction

Chronic obstructive pulmonary disease (COPD) is a major global health burden and remains a leading cause of morbidity and mortality worldwide [1]. It is primarily driven by chronic exposure to noxious particles and gases, most commonly tobacco smoke, but also biomass fuels, occupational irritants, and ambient air pollution [2]. These exposures induce persistent airway and parenchymal inflammation, leading to emphysema, small airway fibrosis, and bronchiolitis, which collectively impair expiratory airflow and promote dynamic hyperinflation [3,4]. Disease progression is frequently punctuated by acute exacerbations, characterized by intensified respiratory symptoms and systemic inflammation, which accelerate lung function decline, impair quality of life, and increase healthcare utilization and mortality [5,6].

Beyond pulmonary impairment, COPD is increasingly recognized as a systemic disease with significant extra-pulmonary manifestations. Among these, skeletal muscle dysfunction is one of the most clinically relevant, characterized by reduced muscle mass, decreased strength, impaired oxidative capacity, and diminished metabolic flexibility [7,8]. These abnormalities are strong predictors of disability, hospitalization, and mortality, independent of the degree of airflow limitation, and represent a central component of the systemic burden of COPD [3,9]. Within this spectrum, sarcopenia represents the clinically defined manifestation of muscle dysfunction [10].

Sarcopenia is now recognized as a muscle disease characterized by impaired muscle strength, quantity, and function [10,11]. According to the revised European Working Group on Sarcopenia in Older People (EWGSOP2), low muscle strength is the primary diagnostic criterion, supported by measures of muscle quantity and physical performance [10,12]. Sarcopenia is highly prevalent in older adults and in individuals with chronic diseases, and is associated with increased risks of falls, disability, hospitalization, and mortality [13,14]. Although these conditions may overlap in COPD, they represent distinct pathophysiological entities with different therapeutic implications [15].

Frailty, in contrast, represents a broader clinical syndrome characterized by diminished physiological reserve and increased vulnerability to stressors [16,17]. The physical frailty phenotype includes five criteria unintentional weight loss, weakness, exhaustion, slow gait speed, and low physical activity of which weakness and slowness are directly linked to skeletal muscle impairment [18,19]. This model reflects “primary frailty,” which is predominantly age-related. However, in chronic diseases such as COPD, a form of “secondary frailty” emerges, driven by disease-specific pathophysiological processes including systemic inflammation, hypoxemia, and physical inactivity [20,21].

Sarcopenia is increasingly recognized as a central biological substrate and major contributor to physical frailty [22]. Loss of muscle strength and mass directly contributes to core frailty components, particularly weakness, reduced mobility, and decreased activity levels [23]. This mechanistic overlap explains the frequent coexistence of these conditions and supports the concept that sarcopenia represents an early and potentially modifiable stage in the trajectory toward frailty [24]. In COPD, this relationship is further amplified by systemic factors such as chronic inflammation, oxidative stress, anabolic resistance, mitochondrial dysfunction, hormonal alterations, and recurrent exacerbations, all of which accelerate muscle catabolism and functional decline [8,25].

Nutritional impairment represents a critical and often under-recognized driver of both sarcopenia and frailty in COPD [26]. Patients frequently experience anorexia, early satiety due to lung hyperinflation, dysphagia, systemic inflammation, and increased resting energy expenditure, particularly during exacerbations [27,28]. These factors contribute to chronic energy imbalance, inadequate protein intake, and micronutrient deficiencies [29]. At the molecular level, malnutrition exacerbates anabolic resistance by impairing IGF-1/Akt/mTOR signaling, while promoting proteolysis via FOXO-mediated activation of ubiquitin–proteasome pathways. Concurrent mitochondrial dysfunction and oxidative stress further exacerbate impairments in muscle integrity and bioenergetic function. Importantly, nutritional deficits also attenuate the effectiveness of pulmonary rehabilitation, impair immune function, and contribute to increased morbidity and mortality risk [30,31,32].

Despite increasing recognition of the interrelationship between COPD, sarcopenia, frailty, and malnutrition, important knowledge gaps remain. Previous reviews have often addressed these conditions separately or focused on specific clinical or mechanistic aspects, with limited integration of molecular pathways, nutritional status, and therapeutic interventions [25]. Malnutrition remains underrecognized as a key modifiable factor linking disease mechanisms to treatment strategies, despite evolving consensus definitions and growing interest in nutrition-based interventions [33].

This review provides an updated synthesis of the relationship between sarcopenia and frailty in COPD, emphasizing the mechanistic role of malnutrition. By integrating current evidence on molecular pathways, biomarkers, and targeted nutritional and rehabilitative interventions, it aims to clarify underlying mechanisms, identify clinically relevant targets, and support more effective multidisciplinary management approaches.

## 2. Methodology

### 2.1. Literature Search Strategy

A structured literature search was conducted in PubMed, Embase, Scopus, and Web of Science to identify relevant studies published between January 2000 and June 2025. The search strategy combined Medical Subject Headings (MeSH) and free-text terms related to chronic obstructive pulmonary disease (COPD), sarcopenia, frailty, nutrition, mechanistic pathways, and biomarkers, using Boolean operators to optimize sensitivity and specificity.

Search terms included disease-specific keywords (e.g., “COPD,” “chronic bronchitis,” “emphysema”); muscle-related terms (e.g., “sarcopenia,” “muscle wasting”); frailty descriptors; and key mechanistic domains such as inflammation, oxidative stress, mitochondrial dysfunction, anabolic resistance, and nutrition (e.g., malnutrition, dietary intake). Additional terms captured biomarker-related literature, including diagnostic, imaging, and functional markers.

### 2.2. Eligibility Criteria

Eligible studies included original research articles, systematic reviews, and meta-analyses focusing on COPD populations and addressing mechanisms, nutritional aspects, or biomarkers related to sarcopenia and/or frailty. Studies published in English within the specified timeframe were included. To support mechanistic interpretation, relevant preclinical studies (animal and in vitro models) were also considered where human data were limited, although these were interpreted cautiously and clearly distinguished from clinical evidence. Exclusion criteria comprised studies not directly addressing sarcopenia or frailty in COPD, conference abstracts, editorials, and case reports without primary data.

### 2.3. Study Selection

**Figure 1:**
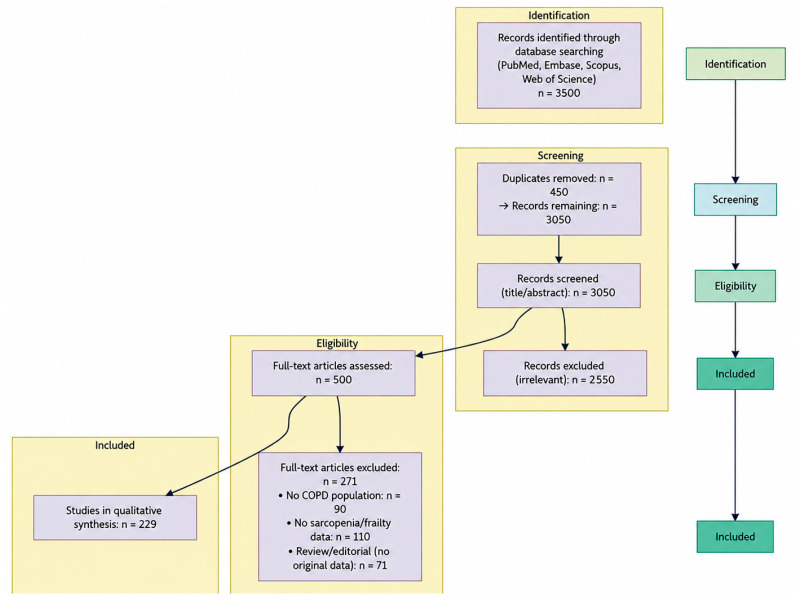
PRISMA Flow Diagram of the Study Selection Process.

Study selection followed PRISMA-informed principles. After removal of duplicates, two independent reviewers screened titles and abstracts for relevance. Full-text articles were subsequently assessed against predefined eligibility criteria. Discrepancies were resolved through discussion or consultation with a third reviewer (Figure 1).

### 2.4. Data Extraction

Data was extracted systematically, including study characteristics (author, year, study design, and sample size) and key findings related to mechanistic pathways (e.g., inflammatory, oxidative, metabolic, and mitochondrial processes) and biomarkers. Biomarkers were categorized as muscle-derived (e.g., myostatin, creatine kinase), systemic (e.g., CRP, IL-6, TNF-α), or imaging/functional (e.g., CT- or MRI-derived muscle mass, handgrip strength, and gait speed).

### 2.5. Quality Assessment

Methodological quality was appraised using the Newcastle–Ottawa Scale (NOS) for observational studies and the Critical Appraisal Skills Programme (CASP) checklist for systematic reviews. These tools were used to evaluate study selection, comparability, and outcome/exposure assessment, providing context for interpreting the strength of the evidence.

### 2.6. Data Synthesis and Review Approach

Given the heterogeneity of study designs, populations, and outcome measures, a narrative synthesis approach was adopted. This review should therefore be considered a systematically informed narrative review rather than a formal systematic review or meta-analysis.

The synthesis prioritizes integration of mechanistic pathways, nutritional factors, and biomarker evidence, with explicit differentiation between preclinical, associative clinical, and interventional data. Emphasis is placed on identifying interactions between pathways and areas where causal inference remains limited, in order to coherent and clinically relevant framework for understanding sarcopenia and frailty in COPD.

## 3. Mechanistic Pathways of Sarcopenia and Frailty in COPD

Mechanistic insights into COPD-related sarcopenia and frailty are derived mainly from observational studies and preclinical models, while relatively few findings have been confirmed in longitudinal or interventional human studies. Accordingly, evidence is classified as preclinical, observational, or interventional, with the latter providing the strongest support for causal inference. Where evidence is limited to preclinical or observational data, we use cautious terms such as “is associated with,” “has been linked to,” or “may contribute to.” Thus, many proposed mechanisms remain provisional and require validation in prospective human studies [34].

### 3.1. Inflammation

Chronic obstructive pulmonary disease is characterized by persistent pulmonary and systemic inflammation that contributes to skeletal muscle dysfunction and sarcopenia [35]. Elevated circulating cytokines, particularly tumor necrosis factor-alpha (TNF-α) and interleukin-6 (IL-6), are consistently associated with reduced muscle mass and lower handgrip strength in patients with COPD and muscle wasting [36]. Observational and biopsy-based studies suggest that systemic inflammation may promote muscle atrophy through several interconnected mechanisms. Elevated cytokine levels are associated with increased hepatic amino acid diversion toward acute-phase protein synthesis [37], while muscle biopsies from patients with greater inflammatory burden show upregulation of ubiquitin–proteasome pathway components, including Atrogin-1, MuRF-1, and NEDD4 [38]. Reduced circulating concentrations of anabolic hormones, including testosterone, insulin-like growth factor-1 (IGF-1), and dehydroepiandrosterone (DHEA), have also been reported in COPD and are inversely associated with IL-6 levels [39].

Together, these findings support a shift toward a net catabolic state. However, causality remains uncertain because muscle wasting itself may contribute to systemic inflammatory activation [40]. Experimental studies implicate TNF-α in the activation of catabolic signaling pathways and impaired myogenic differentiation, thereby promoting muscle loss. However, interventional evidence demonstrating that TNF-α inhibition preserves muscle mass in COPD is lacking [41]. Similarly, IL-6 has been associated with increased proteolysis and myostatin expression in observational studies, but whether pharmacological IL-6 inhibition can prevent or reverse muscle decline in COPD remains unknown [42]. These pathways should therefore be considered biologically plausible rather than conclusively causal in human disease. Inflammation and nutritional status are closely interconnected in COPD, creating a bidirectional cycle that exacerbates metabolic dysfunction [43].

Protein–energy malnutrition may increase inflammatory activity and suppress anabolic signaling, whereas pro-inflammatory cytokines reduce appetite, impair gastrointestinal function, and worsen nutritional status. Diets deficient in antioxidants and omega-3 fatty acids may further sustain oxidative stress and inflammatory activation, accelerating muscle catabolism [44]. Conversely, nutritional interventions, particularly leucine-rich protein, omega-3 fatty acids, and vitamin D supplementation, may attenuate TNF-α and IL-6 activity and partially reduce systemic inflammation, although robust COPD-specific interventional evidence remains limited [45,46]. Beyond its direct catabolic effects, systemic inflammation interacts with nutritional and metabolic pathways to promote mitochondrial dysfunction and anabolic resistance. Collectively, these interrelated mechanisms may perpetuate a self-reinforcing cycle of muscle loss and functional decline in COPD [35,47]. These mechanistic proposals should therefore be regarded as well-supported hypotheses rather than established causal pathways pending confirmatory interventional evidence (Table 1).

### 3.2. Disrupted Anabolic–Catabolic Signaling and Impaired Muscle Regeneration

In COPD, skeletal muscle homeostasis is disrupted by coordinated alterations in anabolic and catabolic signaling, shifting protein turnover toward degradation and muscle atrophy [51]. Myostatin (MSTN), a member of the transforming growth factor-β (TGF-β) superfamily, is a potent negative regulator of muscle growth and is elevated in COPD, where it may contribute to sarcopenia progression [52]. In contrast, insulin-like growth factor-1 (IGF-1) promotes anabolic signaling and muscle protein synthesis. Accordingly, the balance between MSTN-mediated inhibition and IGF-1-driven anabolic activity is critical for maintaining skeletal muscle mass in COPD [53].

The IGF-1/PI3K/Akt/FoxO pathway is a central regulator of muscle protein turnover. Reduced Akt activity promotes nuclear translocation and activation of FoxO transcription factors, which upregulate muscle-specific ubiquitin ligases, including MAFbx and MuRF1, thereby accelerating proteolysis [54,55]. Conversely, inhibition of FoxO signaling suppresses these catabolic pathways and helps preserve muscle mass. Pulmonary rehabilitation has been shown to favorably modulate this signaling network by enhancing IGF-1 activity and attenuating MSTN-mediated inhibitory signaling, thereby supporting muscle maintenance and function [56].

Impaired regenerative capacity also contributes to COPD-related sarcopenia. Skeletal muscle regeneration depends on satellite cells, resident muscle stem cells that mediate repair and adaptation following injury [57]. Under physiological conditions, mechanical and metabolic stimuli activate satellite cells, triggering their proliferation and differentiation into mature myofibers. In COPD, however, chronic systemic inflammation, oxidative stress, hypoxemia, and nutritional deficiency impair satellite cell activation and differentiation [51].

Experimental evidence indicates that pro-inflammatory cytokines, including TNF-α and IL-6, inhibit myogenic differentiation and reduce regenerative potential, whereas oxidative stress promotes cellular senescence and impairs proliferative capacity [58]. Mitochondrial dysfunction further compromises satellite cell function by limiting ATP availability for energy-dependent regenerative processes. In addition, reduced IGF-1 signaling and anabolic resistance diminish satellite cell responsiveness to nutritional and exercise stimuli [59].

Collectively, these alterations create an imbalance between muscle injury and repair, whereby regenerative capacity fails to compensate for ongoing proteolysis. The resulting deficit contributes to progressive muscle wasting and functional decline in COPD-related sarcopenia and frailty [57]. Although emerging preclinical and observational evidence supports these mechanisms, longitudinal human data remain limited, and further research is needed to clarify the causal contribution of satellite cell dysfunction to COPD-associated muscle pathology. These alterations in protein turnover are further compounded by mitochondrial dysfunction and impaired regenerative capacity, which together limit the ability of skeletal muscle to adapt to anabolic stimuli [60] (Table 2).

### 3.3. Oxidative Stress

Converging evidence from preclinical models, muscle biopsy studies, and observational cohorts implicates oxidative stress in skeletal muscle dysfunction in COPD, although establishing a definitive causal role in humans remains challenging [64]. Excessive reactive oxygen and nitrogen species (RONS), generated by hypoxia, chronic smoke-induced inflammation, increased ventilatory demand, and mitochondrial by-products, are associated with lipid peroxidation, protein oxidation and nitration, and DNA damage in peripheral muscle tissue [65]. Biopsy studies of the vastus lateralis in patients with moderate-to-severe COPD have demonstrated elevated levels of 4-hydroxy-2-nonenal (4-HNE), lipofuscin, and protein tyrosine nitration, findings that correlate with reduced muscle strength and endurance [66].

However, the cross-sectional nature of these studies precludes determination of whether elevated RONS drive functional decline, arises because of reduced physical activity and mitochondrial deconditioning, or reflect a bidirectional relationship. This limitation also applies to the downstream mechanisms discussed below, which are supported by in vitro and animal studies but require validation in prospective human investigations [67]. Beyond impairing bioenergetics, mitochondrial dysfunction amplifies inflammatory signaling and promotes anabolic resistance, linking cellular energy deficits to broader metabolic dysregulation. In addition to impairing bioenergetics, mitochondrial dysfunction reinforces inflammatory signaling and contributes to anabolic resistance, thereby linking cellular energy failure to systemic metabolic dysregulation [68].

#### 3.3.1. Increased Skeletal Muscle Protein Degradation

Elevated ROS activates multiple stress-responsive signaling pathways that promote proteolysis and autophagy, contributing to progressive muscle wasting [69]. Autophagy, normally required for protein turnover, becomes pathologically upregulated through dysregulated AMPK activation and suppressed Akt/mTORC1 signaling, while proteolytic enzymes including calpain and caspase-3 further accelerate protein breakdown, contributing to muscle atrophy and functional decline [70] (Table 3).

#### 3.3.2. Mitochondrial Damage

Mitochondria, the main source of ROS, are highly vulnerable to oxidative stress. Excess ROS and RNS promote mitochondrial membrane permeability transition, lipid peroxidation, and release of apoptogenic proteins such as cytochrome c and apoptosis-inducing factor, initiating mitochondria-dependent apoptosis [71]. Oxidative damage to respiratory chain complexes I, II, and IV impairs enzyme activity, disrupts oxidative phosphorylation, and compromises mitochondrial DNA integrity, ultimately leading to bioenergetic dysfunction [72] (Table 3).

#### 3.3.3. Impaired Muscle Filament Function

High intracellular ROS and RNS levels impair Na^+^/K^+^-ATPases, calcium pumps, and other ion transporters, thereby disrupting membrane excitability and excitation–contraction coupling. These alterations reduce actin–myosin cross-bridge efficiency, ATP hydrolysis, and shortening velocity, resulting in impaired contractile performance [73] (Table 3).

**Table 3:** Evidence on oxidative stress and skeletal muscle dysfunction in COPD.

Author, Year	Design	Population	COPD Severity	Mechanism	Comparator	Outcomes	Key Findings	Stats	Limitations
Rabinovich et al., 2007 [74]	*Cross-sectional biopsy*	*n* = 29 (22 COPD, 7 controls)	Stable COPD (BMI-stratified)	Mitochondrial dysfunction (ACR, UCP3, redox status)	Controls; COPD with normal BMI	ACR; UCP3; GSH/GSSG; fiber type; capillarity; strength; endurance	ACR markedly reduced in low-BMI COPD; correlated with PaO_2_ and endurance, inversely with lactate; ↓ UCP3 suggests impaired mitochondrial protection	ACR: 2.2 vs. 5.3 vs. 8.2 (BMIL vs. BMIN vs. controls); correlations significant (NR)	Small sample; male-only; cross-sectional; GOLD severity NR; causality unclear
Reid, 2008 [75]	Narrative review	Experimental + human studies	Not COPD-specific	ROS-induced contractile dysfunction; antioxidant (NAC) effects	Saline/untreated controls	Force; Ca^2+^ sensitivity; fatigue; glutathione; K^+^ handling	ROS reduce Ca^2+^ sensitivity → fatigue; NAC delays fatigue across models (↑ endurance and force)	Cycling: +26% time to fatigue; inspiratory loading: +62%; handgrip: +30%; muscle force: +15%	Not COPD-specific; largely experimental; limited clinical translation

Abbreviations: ACR: aconitase activity ratio; UCP3: uncoupling protein 3; GSH: reduced glutathione; GSSG: oxidized glutathione; PaO_2_: arterial oxygen partial pressure; ROS: reactive oxygen species; NAC: N-acetylcysteine; Ca^2+^: calcium ion; NR: not reported. BMIL, COPD patients with low body mass index; BMIN, COPD patients with normal body mass index; ↑ increase, ↓ decrease.

### 3.4. Disuse and Physical Inactivity

In COPD, progressive airflow obstruction, dyspnea, and hyperinflation substantially reduce physical activity, promoting muscle atrophy, fiber-type shifts, capillary rarefaction, and impaired oxidative enzyme capacity. These adaptations contribute to losses in muscle mass, strength, and endurance, thereby accelerating functional decline [76]. Although physical inactivity reduces the proportion of type I muscle fibers by up to one-third in healthy individuals, patients with COPD exhibit more pronounced structural and metabolic abnormalities, indicating the contribution of disease-specific mechanisms beyond deconditioning alone [77]. Muscle wasting is driven by increased proteolysis, suppressed anabolic signaling, and reduced mitochondrial oxidative capacity [78]. In addition, oxidative stress impairs satellite cell function and mitochondrial integrity, further limiting muscle repair and regenerative capacity [79].

### 3.5. Hypoxemia

Chronic hypoxemia, a common consequence of persistent airflow limitation and alveolar-capillary destruction, further contributes to skeletal muscle dysfunction in COPD [66]. Sustained hypoxia promotes inflammation, oxidative stress, impaired myogenic differentiation, and shifts from oxidative (type I and IIa) to glycolytic (type IIb) muscle fibers. Hypoxemic patients with COPD exhibit a lower proportion of type I fibers in peripheral muscles, which correlate closely with arterial oxygen tension. Together with systemic inflammation and nutritional impairment, these alterations perpetuate a cycle of deconditioning, muscle loss, and declining functional capacity [80,81] (Table 4).

### 3.6. Glucocorticoid-Induced Muscle Atrophy

Glucocorticoids, frequently administered for COPD exacerbations and advanced disease, induce muscle atrophy by suppressing protein synthesis and promoting catabolic signaling [83]. They inhibit IGF-I expression and its downstream phosphorylation of 4E-BP1 and S6K1, impair PI3K/Akt signaling through IRS-1 degradation and PI3K dysregulation, and upregulate REDD1, thereby inhibiting mTOR activity and further reducing translational efficiency [84] (Table 5).

### 3.7. Nutritional Deficiency and Energy Imbalance

Nutritional deficiencies affect 20–70% of patients with COPD and contribute significantly to sarcopenia, reduced exercise capacity, and impaired quality of life [25]. Energy deficiency activates AMP-activated protein kinase (AMPK), suppresses mTORC1 signaling, and stimulates autophagy through ULK1 phosphorylation. Simultaneously, catabolic pathways enhance ubiquitin–proteasome activity and autophagy, accelerating muscle protein degradation [30,86] (Table 6).

### 3.8. Integrated Pathogenic Network Linking Inflammation, Malnutrition, Mitochondrial Dysfunction, and Impaired Muscle Regeneration in COPD

Current evidence indicates that sarcopenia and frailty in COPD arise from interconnected pathological processes rather than isolated mechanisms [25]. Chronic inflammation, nutritional deficiency, mitochondrial dysfunction, anabolic resistance, impaired muscle regeneration, hypoxemia, and physical inactivity interact through bidirectional, self-reinforcing pathways that progressively impair skeletal muscle structure and function [89,90].

Chronic systemic inflammation is a key upstream driver. Elevated TNF-α, IL-6, and other pro-inflammatory mediators stimulate muscle protein degradation while suppressing anabolic signaling required for muscle maintenance and repair [91]. These cytokines also promote anorexia, increased resting energy expenditure, and metabolic dysregulation, contributing to protein–energy malnutrition [92]. Conversely, malnutrition may worsen inflammation through impaired antioxidant defenses, reduced immune resilience, and increased oxidative stress, establishing a bidirectional inflammation–nutrition axis [93].

Mitochondrial dysfunction serves as a key convergence point linking inflammation, hypoxemia, cigarette smoke exposure, physical inactivity, and inadequate nutrient intake. These factors impair mitochondrial biogenesis, respiratory chain function, and ATP production [94]. Dysfunctional mitochondria generate excess reactive oxygen species, amplifying inflammatory signaling and oxidative damage, while reduced ATP availability impairs protein synthesis, contractile performance, and adaptive responses to exercise [95].

Together, these abnormalities contribute to anabolic resistance. Persistent inflammation suppresses IGF-1/Akt/mTOR signaling, nutrient deficiency limits substrate availability, and mitochondrial dysfunction restricts the energy required for protein synthesis [96]. Consequently, skeletal muscle exhibits a diminished anabolic response to dietary protein and exercise, increasing the anabolic stimulus required to preserve muscle mass and function [97].

Muscle loss is further accelerated by impaired regeneration. Chronic inflammation, oxidative stress, and reduced anabolic signaling compromise satellite cell activation, proliferation, and differentiation [98]. Experimental studies indicate that inflammatory cytokines impair myogenic differentiation, whereas mitochondrial dysfunction limits the bioenergetic support required for effective regeneration [99]. As regenerative capacity declines, protein degradation progressively exceeds repair mechanisms, leading to muscle wasting [100].

Physical inactivity and hypoxemia further amplify this pathogenic network. Reduced activity promotes mitochondrial deconditioning, anabolic resistance, and loss of oxidative muscle fibers, whereas chronic hypoxia increases oxidative stress, inflammatory activation, and metabolic inefficiency [77]. These changes diminish exercise tolerance and physical performance, reinforcing inactivity and perpetuating functional decline [47].

Collectively, inflammation, malnutrition, mitochondrial dysfunction, anabolic resistance, impaired regeneration, hypoxemia, and physical inactivity form an integrated pathogenic network driving sarcopenia and frailty in COPD [101]. This framework supports multimodal therapeutic strategies combining nutritional optimization, exercise-based rehabilitation, and interventions targeting systemic inflammation and mitochondrial function rather than addressing individual pathogenic pathways in isolation [102].

### 3.9. The Role of Nutrition in COPD-Related Sarcopenia and Frailty

Nutrition plays a central role in maintaining skeletal muscle mass, metabolic function, and physical resilience, and its impairment represents a key accelerator of sarcopenia and frailty in COPD [103]. Between 20–70% of COPD patients exhibit some degree of malnutrition, driven by anorexia, dyspnea, systemic inflammation, low-grade acidosis, early satiety, dysphagia, and medication effect [27]. These disturbances intersect with COPD-related catabolic pathways, leading to accelerated muscle loss, reduced exercise capacity, and increased vulnerability to exacerbations [25].

#### 3.9.1. Macronutrient Requirements, Protein Anabolism, and Anabolic Resistance

Protein intake is a key determinant of skeletal muscle preservation; however, its effectiveness in COPD is limited by anabolic resistance, defined as a blunted muscle protein synthesis (MPS) response to a given protein or amino acid stimulus. This phenomenon results from the combined effects of systemic inflammation, elevated muscle proteolysis, mitochondrial dysfunction, impaired amino acid sensing by mTORC1, and physical inactivity [97,104]. Consequently, patients with COPD and sarcopenia require higher protein intakes than healthy age-matched individuals to achieve comparable anabolic responses [60].

Protein dosing: Current clinical and mechanistic evidence supports protein intakes of 1.2–1.5 g/kg/day in stable COPD with sarcopenia and up to 1.5–2.0 g/kg/day during acute exacerbations or recovery periods characterized by increased catabolic stress [104]. These recommendations exceed the standard dietary allowance of 0.8 g/kg/day and reflect the degree of anabolic resistance present. Protein intake should ideally be distributed across three to four meals providing ≥0.4 g/kg per meal to maximize mTORC1 activation and MPS, as large single boluses do not appear superior for overcoming anabolic resistance [105].

Leucine enrichment: Leucine is a potent activator of mTORC1 through the Rag GTPase–Regulator pathway and stimulates anabolic signaling independently of total amino acid availability. Leucine-rich protein sources, including whey, casein, dairy products, soy isolate, and legumes, may partially overcome anabolic resistance by enhancing S6K1 and 4E-BP1 phosphorylation, thereby promoting ribosomal biogenesis and MPS despite reduced Akt activity [106]. β-Hydroxy-β-methylbutyrate (HMB), a leucine metabolite, has shown potential to reduce proteolysis and improve lean mass at doses of 3 g/day in undernourished or cachectic populations, although COPD-specific evidence remains limited [107].

Timing relative to rehabilitation: Consuming protein within 0–2 hours after resistance or endurance exercise enhances MPS by augmenting exercise-induced mTORC1 activation and satellite cell recruitment [108]. Accordingly, patients participating in pulmonary rehabilitation should be encouraged to consume a protein-containing meal or supplement during this post-exercise period. Pre-exercise protein ingestion (30–60 min before training) may increase amino acid availability during exercise, although evidence supporting superiority over post-exercise intake in COPD remains inconclusive [109].

Duration: Intervention studies have ranged from 8 weeks to 24 months, with most improvements in muscle mass and strength observed within 8–12 weeks of combined nutritional and exercise interventions. Continued dietary optimization appears necessary, as gains in muscle mass may diminish following discontinuation of protein supplementation in the presence of persistent anabolic resistance [110].

Energy targets: Resting energy expenditure is elevated by 10–30% in many patients with COPD owing to increased work of breathing, systemic inflammation, and β-agonist therapy. Energy intakes of 30–45 kcal/kg/day are supported by clinical guidelines and should be individualized according to disease severity, BMI, and physical activity level [26,111]. During acute exacerbations, particularly those requiring hospitalization, energy requirements may increase further, and enteral supplementation should be considered when oral intake remains inadequate for more than 48–72 h [112].

Macronutrient composition and ventilatory efficiency: Because carbohydrate oxidation generates more carbon dioxide than fat oxidation, excessive carbohydrate intake may worsen CO_2_ retention and dyspnea in patients with severe airflow limitation (FEV_1_ < 50% predicted) or hypercapnia. Replacing refined carbohydrates with monounsaturated and polyunsaturated fats while maintaining adequate caloric intake may improve ventilatory efficiency in selected patients [113]. However, very low-carbohydrate diets should be avoided in individuals receiving corticosteroids or those with diabetes, in whom glycemic monitoring remains essential [114].

Contraindications and individual variability: High-protein supplementation is generally safe but requires caution in patients with stage 3–4 chronic kidney disease, where protein restriction (0.6–0.8 g/kg/day) may be necessary. Significant hepatic dysfunction may similarly limit aggressive protein supplementation [114]. Anabolic responsiveness varies considerably among individuals and may be influenced by genetic factors, gut microbiome composition, systemic inflammatory burden, and medication exposure, particularly corticosteroid use. Although pharmacogenomic and metabolomic approaches may eventually support personalized protein prescriptions, these strategies remain investigational and are not yet part of routine clinical practice [115].

#### 3.9.2. Micronutrients and Muscle Health

Chronic obstructive pulmonary disease is frequently associated with micronutrient deficiencies resulting from inadequate dietary intake, systemic inflammation, polypharmacy, and increased metabolic demands. These deficiencies may contribute to skeletal muscle dysfunction through nutrient-specific mechanisms [46].

Vitamin D deficiency (25(OH)D < 50 nmol/L), reported in 60–80% of patients with COPD, has been linked to impaired muscle function through effects on calcium-dependent contractile signaling, IGF-1/Akt pathway activation, myogenic differentiation, and mitochondrial respiration [116]. Longitudinal studies associate deficiency with accelerated declines in muscle strength and increased sarcopenia risk. Supplementation appears most beneficial in individuals with low baseline vitamin D levels, producing modest improvements in muscle strength but inconsistent effects on muscle mass. These findings support routine assessment and correction of deficiency [117].

Omega-3 fatty acids, particularly eicosapentaenoic acid (EPA) and docosahexaenoic acid (DHA), may modulate inflammatory signaling, enhance anabolic sensitivity, and reduce proteolysis [118]. Studies in older adults suggest that supplementation (2–4 g/day for 12–24 weeks) produces modest improvements in muscle mass and physical performance. However, evidence in COPD remains limited and heterogeneous, preventing firm conclusions regarding clinical efficacy [119,120].

Antioxidant nutrients, including vitamins C and E and carotenoids, have attracted interest because oxidative stress contributes to COPD-related muscle dysfunction [121]. Although observational studies associate higher antioxidant intake with better muscle outcomes, randomized trials of high-dose supplementation have yielded inconsistent results and may attenuate exercise-induced adaptations. Current evidence therefore favors antioxidant-rich dietary patterns over routine high-dose supplementation [122].

B vitamins, including thiamine, riboflavin, vitamin B_6_, vitamin B_12_, and folate, are essential for mitochondrial energy metabolism and amino acid utilization. Deficiencies, potentially exacerbated by comorbidities and medication use, have been associated with fatigue and impaired muscle function. However, COPD-specific interventional evidence remains limited, and supplementation should be guided by documented deficiency and individual risk factors [123].

Magnesium deficiency is common in COPD, particularly among patients receiving diuretics, β_2_-agonists, or corticosteroids. Given its role in neuromuscular excitability and muscle contraction, deficiency may contribute to muscle dysfunction. Although observational studies report associations with disease severity and inflammation, supplementation trials have shown inconsistent functional benefits; therefore, routine supplementation is not recommended without confirmed deficiency [124].

Iron deficiency affects approximately 30–47% of patients with COPD and may impair muscle function through its roles in oxygen transport and mitochondrial respiration. Both anemic and non-anemic iron deficiency have been associated with reduced exercise capacity and poorer responses to pulmonary rehabilitation. Emerging evidence suggests that iron repletion, particularly via intravenous administration, may improve exercise tolerance and quality of life, supporting routine assessment and targeted management of iron deficiency in COPD [125].

#### 3.9.3. Oral Nutritional Supplements (ONS): Dosing, Duration, and Clinical Indications

Oral nutritional supplements (ONS) represent a practical strategy to increase energy and protein intake in patients with COPD who are unable to meet nutritional requirements through habitual dietary intake alone. Their use is particularly relevant in individuals with malnutrition or nutritional risk, including those with low body mass index, unintentional weight loss, reduced fat-free mass, cachexia, or persistently inadequate dietary intake despite nutritional counselling [123,126].

Current evidence supports the use of energy- and protein-enriched ONS, particularly in underweight and nutritionally depleted patients with COPD [123,127]. Randomized trials and systematic reviews indicate that supplementation providing approximately 400–600 kcal/day in addition to habitual intake for 8–16 weeks can increase body weight and fat-free mass, with the greatest benefits observed in patients with more severe nutritional impairment [128,129]. Improvements in muscle strength, exercise capacity, physical performance, and health-related quality of life have also been reported, although findings are less consistent and may be influenced by baseline nutritional status, study design, and concurrent pulmonary rehabilitation [129,130].

Several studies have evaluated disease-specific formulations enriched with protein and other potentially anabolic or anti-inflammatory nutrients [131,132]. Such approaches are biologically plausible given the presence of anabolic resistance, systemic inflammation, and accelerated muscle protein breakdown in COPD; however, the available evidence remains limited [64]. Many studies have enrolled heterogeneous patient populations, combined nutritional supplementation with exercise training or pulmonary rehabilitation, or assessed multi-component interventions, making it difficult to determine the independent effects of individual nutrients on muscle mass, strength, or functional outcomes [109,133].

The duration of supplementation in clinical studies has generally ranged from 8 to 24 weeks. Although formulations containing high-quality protein, leucine-rich amino acids, HMB, and omega-3 polyunsaturated fatty acids have shown potential benefits for body composition, exercise tolerance, and metabolic outcomes in selected COPD populations, the evidence is limited by small sample sizes, short intervention durations, heterogeneous populations, and variability in supplement composition [109,134]. In addition, many studies combined nutritional supplementation with exercise training, making it difficult to distinguish the independent effects of nutrition from those of rehabilitation. Consequently, current evidence is insufficient to support definitive recommendations regarding specific amino acid- or nutrient-enriched formulations in COPD [26].

Combined nutritional supplementation and pulmonary rehabilitation may provide synergistic benefits; however, interpretation remains limited by heterogeneity in study design, supplement composition, and outcome measures. Further well-designed trials are required to clarify the independent and additive effects of nutritional interventions in patients with sarcopenia and frailty [109].

Overall, ONS should be considered part of a comprehensive multidisciplinary strategy including nutritional assessment, individualized dietary counselling, exercise training, and optimization of COPD care. Adequately powered randomized controlled trials in patients with well-defined sarcopenia and frailty are needed to evaluate the independent effects of high-calorie nutritional support and targeted formulations containing leucine, HMB, and omega-3 fatty acids using standardized diagnostic criteria and clinically relevant functional outcomes. Such evidence is required before firm conclusions can be drawn regarding optimal nutritional strategies for sarcopenic COPD populations [34,135].

#### 3.9.4. Nutrition–Exercise Synergy and Integration with Pulmonary Rehabilitation

The complementary roles of nutritional optimization and exercise training in mitigating sarcopenia are well established in older adults and increasingly supported by COPD-specific studies, although high-quality evidence remains limited [25]. Resistance and endurance exercise activate mTORC1 signaling, promote satellite cell recruitment, and stimulate mitochondrial biogenesis; however, these anabolic responses are attenuated in the presence of inadequate protein or energy intake [136].

Malnutrition blunts the acute muscle protein synthesis response to exercise and impairs longer-term adaptations, including increases in muscle cross-sectional area, fiber-type remodeling, and oxidative enzyme capacity. Consequently, inadequate nutritional status may reduce the effectiveness of pulmonary rehabilitation programs [137,138].

Nutritional assessment and targeted support should therefore be integrated into structured pulmonary rehabilitation as a core component of care rather than an optional adjunct. This includes baseline assessment using validated tools such as the Mini Nutritional Assessment (MNA) or a 3-day food diary reviewed by a registered dietitian; individualized energy and protein targets; prescription of oral nutritional supplements for patients with malnutrition, preferably timed after exercise; and body composition assessment using bioelectrical impedance analysis (BIA) or dual-energy X-ray absorptiometry (DXA) at baseline and program completion [46,135]. Current evidence suggests that combined nutrition and exercise interventions provide additive benefits compared with either intervention alone. However, formal interaction analyses demonstrating true synergistic or supra-additive effects are largely lacking in COPD-specific trials, which is important for interpreting treatment effects and guiding future study design [133,139].

### 3.10. Unhealthy Lifestyles

#### 3.10.1. Smoking

Smoking, the primary risk factor for COPD, also contributes directly to skeletal muscle dysfunction. Cigarette smoke impairs oxygen transport and diffusion, disrupts mitochondrial respiration, and increases oxidative stress, leading to reduced ATP synthesis and contractile function [140,141]. Long-term exposure induces neuromuscular junction degeneration, activates MAPK and NF-κB signaling, accelerates myosin degradation, and promotes proteolysis through upregulation of MAFBx, MuRF-1, and ubiquitin-specific protease [142]. Additional mechanisms include impaired Ca^2+^ signaling, ferroptosis via HIF2α, and suppression of Akt-mediated protein synthesis [143]. Smoking cessation improves mitochondrial function, protein synthesis, and muscle integrity in experimental models, suggesting benefits for both lung function and skeletal muscle health [144].

#### 3.10.2. Alcohol

Chronic alcohol intake exacerbates COPD progression and promotes muscle dysfunction through oxidative stress, mitochondrial impairment, impaired regeneration, and fibrosis [145]. Alcohol reduces IGF-1 availability, disrupts mTOR signaling, and activates proteolytic pathways, including MuRF-1 and MAFBx [146]. Evidence of alcoholic myopathy parallels mechanisms of COPD-associated sarcopenia, though the interaction between alcohol use and COPD-related muscle loss remains insufficiently studied. Given that alcohol intake is elevated in some COPD populations and even intermittent binge drinking induces myofiber atrophy and protein damage, abstinence is strongly recommended [147].

#### 3.10.3. Reduced Physical Activity

Dyspnea-driven inactivity in COPD induces muscle fiber atrophy, reduced oxidative capacity, and capillary rarefaction, thereby limiting endurance and strength. Inflammatory and hypoxic conditions, including post-COVID states, exacerbate these changes. Although inactivity drives muscle remodeling, COPD patients exhibit greater impairment than activity-matched controls, indicating additional disease-related mechanisms [47]. Pulmonary rehabilitation combining aerobic and resistance training improves endurance, gait speed, and strength, though some functional deficits may persist [148] (Table 7).

## 4. Cellular and Microenvironmental Contributors

Sarcopenia in COPD results not only from systemic catabolic processes but also from distinct cellular and microenvironmental alterations within skeletal muscle. Although many of these changes overlap with those observed during primary aging, growing evidence indicates that COPD imposes additional disease-specific insults, including chronic hypoxemia, smoke-induced oxidative stress, persistent local inflammation driven by neutrophils and macrophages, and recurrent corticosteroid exposure [8,25]. These factors accelerate muscle wasting and contribute to a phenotype that differs from age-related sarcopenia. Recognizing these COPD-specific mechanisms is essential for the development of targeted, mechanism-based therapeutic strategies [47].

### 4.1. Roles of Satellite Cells

Skeletal muscle regeneration depends on satellite cells (SCs), myogenic precursor cells located beneath the basal lamina whose activation, proliferation, and differentiation are regulated by neural, vascular, hormonal, nutritional, and injury-related signals. In primary aging, SC numbers decline, particularly in type II fibers, and regenerative capacity progressively deteriorates, contributing to age-related sarcopenia [150]. Notably, aged SCs retain considerable intrinsic proliferative potential in vitro, suggesting that impaired regeneration largely reflects an unfavorable systemic and local microenvironment. This concept is supported by parabiosis studies demonstrating partial restoration of SC function following exposure to a youthful systemic milieu [151].

In COPD, SC dysfunction results from mechanisms that overlap with, but also extend beyond, those observed in primary aging, leading to a greater impairment of muscle regenerative capacity. Thériault et al. reported increased SC senescence in the vastus lateralis of patients with severe COPD, characterized by shortened telomeres, elevated p16^INK4a expression, reduced numbers of Pax7-positive SCs, and impaired regenerative potential. Importantly, these abnormalities persisted after adjustment for age, indicating a disease-specific contribution to SC dysfunction [57].

Several COPD-related factors contribute to accelerated SC impairment. Chronic hypoxemia disrupts hypoxia-inducible factor-1α (HIF-1α)-mediated regulation of SC quiescence and activation, impairing oxygen-sensitive signaling required for effective muscle repair [57]. In parallel, persistent systemic and local inflammation characterized by elevated IL-6, TNF-α, and CRP levels, alongside increased infiltration of macrophages and neutrophils into skeletal muscle, suppresses SC activation and promotes premature senescence via NF-κB signaling, with downstream upregulation of myostatin and activin A [91,152]. Myostatin, a potent inhibitor of Akt/mTOR signaling, is upregulated in COPD skeletal muscle and directly inhibits SC proliferation and myoblast differentiation, independently of age-related effects [153]. Cigarette smoke-derived reactive oxygen species further aggravate these processes by inducing DNA damage, accelerating telomere shortening, and promoting p16^INK4a-mediated cell-cycle arrest, collectively reducing the pool of regeneratively competent SCs [154].

These converging mechanisms impair muscle repair and hypertrophic responses, particularly after the acute catabolic stress associated with COPD exacerbations. Evidence from Vogiatzis et al. indicates that pulmonary rehabilitation partially restores myogenic signaling, reflected by increased MyoD and myogenin expression and improved muscle fiber cross-sectional area, suggesting that the SC compartment retains functional plasticity even in advanced disease [155]. However, the magnitude and durability of these adaptations remain limited by the persistent inflammatory and hypoxic milieu, supporting the rationale for combined anabolic, anti-inflammatory, and exercise-based interventions [156].

A key distinction from primary sarcopenia is that SC dysfunction in aging is largely driven by intrinsic cellular alterations and reduced systemic anabolic signaling. In COPD, additional disease-specific stressors including chronic hypoxemia, sustained inflammation, corticosteroid exposure, and smoke-induced genotoxic damage further compromise SC function and regenerative capacity [60]. Therefore, effective therapeutic strategies must target both SC biology and the hostile microenvironment in which these cells reside [157] (Table 8).

### 4.2. Muscle Vasculature

Vascular integrity is essential for skeletal muscle oxygen delivery, nutrient supply, and exercise performance, and its impairment contributes significantly to sarcopenia in both aging and COPD. Although some mechanisms overlap, important disease-specific differences distinguish COPD-associated vascular dysfunction from age-related vascular decline [159].

In primary aging, skeletal muscle blood flow is characterized by impaired endothelium-dependent vasodilation, whereas endothelium-independent vasodilatory pathways are largely preserved [160]. Reduced nitric oxide (NO) bioavailability, driven by decreased endothelial NO synthase (eNOS) activity and increased oxidative inactivation of NO, contributes to impaired vasodilatory signaling, attenuated contraction-induced vasodilation, and diminished rapid-onset vasodilation [161]. Behnke et al. demonstrated reduced skeletal muscle microvascular oxygen pressure during muscle contractions in aged rats, indicating impaired oxygen delivery at the tissue level [162]. In older adults, Donato et al. linked reduced flow-mediated dilation to increased endothelial oxidative stress, while Eskurza et al. showed that acute antioxidant administration partially restores endothelium-dependent vasodilation, supporting a central role for oxidative stress in vascular aging [163,164]. Aging also impairs prostacyclin-mediated vasodilation and reduces functional sympathy, limiting muscle perfusion during exercise, although regular exercise training can partially restore these responses [165].

In COPD, vascular dysfunction is further aggravated by disease-specific mechanisms. Chronic hypoxemia increases reactive oxygen species production through activation of xanthine oxidase and NADPH oxidase pathways within vascular endothelial cells and skeletal muscle, accelerating NO degradation beyond that observed with aging alone [166]. Cigarette smoke exposure impairs vascular function through endothelial injury, inflammation, reduced eNOS expression, and attenuated acetylcholine-mediated vasodilation. COPD is also associated with capillary rarefaction, characterized by reduced functional capillary density not fully attributable to muscle fiber atrophy [167]. Reduced vascular endothelial growth factor (VEGF) contributes to impaired angiogenesis and diminished capillary maintenance in COPD skeletal muscle, increasing diffusion distances and reducing the surface area for oxygen exchange during exercise. This represents a structural vascular remodeling process distinct from the primarily functional vasodilatory impairment observed in aging [168].

Clinically, these vascular abnormalities contribute to exercise intolerance. Iepsen et al. demonstrated that COPD patients exhibit impaired exercise-induced skeletal muscle oxygen delivery, with reduced limb blood flow and muscle oxygenation compared with age-matched controls, independently contributing to exercise limitation [169]. These findings are consistent with the ERS/ATS statement on limb muscle dysfunction in COPD, which highlights peripheral vascular impairment as a key but underrecognized determinant of reduced exercise capacity beyond ventilatory constraints [170].

Therapeutically, aerobic exercise training within pulmonary rehabilitation promotes angiogenesis via VEGF upregulation, partially restoring capillary density and improving skeletal muscle oxygen conductance [171]. Antioxidant strategies may further enhance nitric oxide bioavailability by reducing vascular oxidative stress, although COPD-specific clinical evidence remains limited. In patients with chronic hypoxemia, long-term oxygen therapy may also attenuate hypoxia-driven vascular dysfunction, but its effects on skeletal muscle microvascular remodeling require further investigation (Table 9) [172].

### 4.3. COPD Phenotype, Severity Stratification, and Comorbidities as Modifiers of Sarcopenia Risk and Therapeutic Response

COPD-related sarcopenia and frailty are clinically heterogeneous, rendering uniform nutritional and rehabilitation approaches insufficient. Disease severity, exacerbation burden, age, and comorbidities substantially influence both the risk of muscle dysfunction and the response to intervention, supporting a phenotype-based management strategy [101].

#### 4.3.1. Disease Severity

Disease severity, defined by GOLD spirometric stage and symptom or exacerbation burden, is a major determinant of sarcopenia risk. Advanced COPD (GOLD 3–4) is consistently associated with reduced fat-free mass, impaired muscle strength, and poorer functional performance due to the combined effects of hypoxemia, systemic inflammation, corticosteroid exposure, and physical inactivity [101,174]. However, spirometric impairment alone does not fully capture risk, as clinically relevant muscle dysfunction may also occur in patients with relatively preserved lung function, particularly those with emphysema-predominant disease [175]. These findings underscore the importance of complementing routine pulmonary assessment with body composition evaluation [176]. In severe disease, nutritional strategies should address increased energy expenditure and metabolic stress while accounting for macronutrient composition in patients with hypercapnia [177].

#### 4.3.2. Exacerbation Burden

Frequent exacerbations constitute a high-risk phenotype characterized by accelerated declines in muscle mass and function [178]. Acute inflammatory responses, reduced nutritional intake, physical inactivity, and corticosteroid exposure during exacerbations contribute cumulatively to skeletal muscle deterioration, generating an “exacerbation-related ratchet effect” [42]. Consequently, early nutritional support during acute illness and structured pulmonary rehabilitation following hospital discharge are essential to mitigate long-term functional impairment [179].

#### 4.3.3. Age-Related Vulnerability

Older adults with COPD experience the combined effects of age-related and disease-specific mechanisms of sarcopenia, including anabolic resistance, hormonal decline, mitochondrial dysfunction, and inflammaging [101]. These biological alterations are frequently compounded by multimorbidity, polypharmacy, and social determinants that compromise dietary intake [180]. As a result, higher per-meal protein doses and leucine-rich protein sources may be necessary to achieve adequate anabolic stimulation. Given the elevated risk of falls and fractures in this population, multicomponent exercise programs incorporating resistance, endurance, and balance training are particularly important [181,182].

#### 4.3.4. Comorbidities

Comorbidities further modify sarcopenia pathophysiology and influence treatment feasibility [183]. Chronic kidney disease complicates nutritional management by requiring a balance between adequate protein intake and renal preservation, necessitating individualized protein prescriptions according to disease stage [184]. Type 2 diabetes mellitus exacerbates anabolic resistance through impaired insulin signaling but may respond favorably to resistance training [185]. Heart failure contributes to cachexia through neurohormonal and inflammatory pathways and may restrict nutritional interventions because of fluid limitations [186]. Osteoporosis commonly coexists with sarcopenia, increasing fracture risk and emphasizing the need for integrated bone–muscle protective strategies, including optimization of vitamin D and calcium status and appropriate pharmacological treatment when indicated [187].

#### 4.3.5. Clinical Implications

Collectively, these phenotypic differences support a transition from generalized recommendations toward individualized, risk-stratified care. Integrating nutritional and rehabilitation strategies within a phenotype-based framework may better align intervention intensity with biological and clinical risk, thereby maximizing functional recovery and improving patient outcomes [188]. Table 10 summarizes a phenotype-guided framework integrating nutritional and rehabilitation strategies for sarcopenia and frailty management in COPD [26,189,190].

## 5. Biomarkers of Sarcopenia and Frailty in COPD

### 5.1. Novel Biomarkers

The search for reliable biomarkers of frailty and sarcopenia in COPD is a key research priority, with potential to enhance diagnosis, monitoring, and personalized management. Urinary titin has shown promise as a marker of muscle atrophy in critical illness, while blood-based biomarkers are increasingly investigated for their utility in assessing sarcopenia and disease progression [25,191]. Such biomarkers provide clinically relevant information on muscle mass, strength, and function, supporting earlier detection of sarcopenia in COPD populations. Given the variability in prevalence estimates across studies, the adoption of standardized diagnostic criteria and cut-off values is essential [192,193]. Integrating biomarker evaluation with consensus-based assessments may improve risk stratification and guide individualized therapeutic strategies for COPD-related sarcopenia and frailty [194].

Several biomarkers appear to reflect nutritional status in COPD and may help identify individuals at risk of sarcopenia. Growth differentiation factor-15 (GDF-15), a cytokine elevated during mitochondrial stress and anorexia, correlates with low muscle mass, reduced strength, and weight loss in COPD patients, indicating its relevance as a malnutrition-associated biomarker [195,196]. Similarly, elevated circulating levels of c-terminal agrin fragment-22 (CAF22) indicate neuromuscular junction instability and are higher in patients with reduced protein intake or muscle wasting [197]. These biomarkers may therefore provide valuable insight into the nutritional and metabolic drivers of sarcopenia, supporting early detection and personalized nutritional interventions (Table 11) [198].

### 5.2. Blood Biomarkers

Circulating biomarkers provide important insights into muscle mass, strength, and frailty in COPD, originating from both muscle-derived and systemic sources. Among muscle-derived markers, creatine kinase (CK) is associated with impaired muscle function, while plasma c-terminal agrin fragment-22 (CAF22) has emerged as a reliable predictor of muscle health [198]. Non-muscle biomarkers, including haptoglobin, hemopexin, ceruloplasmin, resistin, and TNF-α, have also been linked to reduced muscle strength and mass, reflecting systemic inflammation and muscle impairment [201]. Although these markers show diagnostic potential, further validation in large, well-designed studies is required to confirm their independent predictive value [202]. Extending beyond COPD, skeletal muscle dysfunction is common in other chronic respiratory diseases. Recent evidence indicates that circulating levels of Dickkopf-3 (Dkk-3), CAF22, and specific microRNAs (miR-21, miR-133, miR-134a, miR-206) are significantly associated with reduced hand-grip strength, gait speed, and appendicular skeletal muscle mass in patients with COPD, asthma, and pulmonary tuberculosis [203,204]. Collectively, these findings highlight the potential of integrated biomarker panels to improve the detection and monitoring of sarcopenia across respiratory diseases (Table 12) [205].

A representative cohort of COPD patients (*n* = 352) and their body composition characteristics are shown in Table 13. An in-depth understanding of body composition alterations is essential to elucidate the underlying mechanisms and to identify potential therapeutic strategies aimed at improving both body composition and overall survival (Table 13).

The cohort showed elevated mean BMI and body fat percentage consistent with overweight status, alongside reduced skeletal muscle proportion, reflecting sarcopenic tendencies commonly observed in COPD. Resting metabolic rate was moderately decreased relative to reference norms, indicating lowered basal energy expenditure. The variability across patients (as shown by SD values) highlights heterogeneity in nutritional and metabolic profiles before initiation of pulmonary rehabilitation.

## 6. Discussion

Chronic obstructive pulmonary disease (COPD) is increasingly recognized as a systemic disorder in which skeletal muscle dysfunction represents a central determinant of morbidity, functional decline, and mortality [8,175]. While previous sections have examined individual mechanistic pathways, the present synthesis highlights that COPD-related sarcopenia and frailty arise not from isolated abnormalities but from a highly interconnected pathogenic network linking inflammation, nutritional impairment, mitochondrial dysfunction, and impaired muscle regeneration [103,208].

At the core of this network lies chronic systemic inflammation, which acts as a primary upstream driver. Persistent elevations in inflammatory mediators promote proteolytic activation through ubiquitin–proteasome and autophagy–lysosomal pathways while simultaneously impairing anabolic signaling [209,210]. Importantly, inflammation does not operate in isolation; it is tightly coupled with nutritional status and metabolic regulation. In COPD, reduced dietary intake, increased resting energy expenditure, and recurrent exacerbations create a state of chronic energy deficit that amplifies anabolic resistance and accelerates muscle catabolism [111,123]. This interaction between inflammation and malnutrition represents a critical, yet often underappreciated, axis driving disease progression [211].

Mitochondrial dysfunction emerges as a central downstream consequence of this inflammatory–nutritional imbalance [212]. Impaired oxidative phosphorylation, reduced ATP production, and increased reactive oxygen species generation contribute not only to muscle fatigue but also to further activation of proteolytic pathways, thereby reinforcing muscle wasting [30]. Notably, mitochondrial abnormalities in COPD appear to be more pronounced than those observed in normal aging, likely reflecting the combined effects of hypoxemia, oxidative stress from smoking exposure, and physical inactivity [213]. This distinction underscores the importance of considering COPD-specific mechanisms rather than extrapolating solely from geriatric models of sarcopenia [35].

Impaired regenerative capacity further compounds muscle deterioration. Satellite cell dysfunction, influenced by inflammatory signaling and altered metabolic environments, limits the ability of skeletal muscle to repair and adapt [214]. Concurrent vascular abnormalities reduce oxygen and nutrient delivery, exacerbating local hypoxia and metabolic stress [215]. These processes are coordinated at the transcriptional level, where dysregulated gene expression programs disrupt the balance between anabolic and catabolic pathways [216]. Rather than acting independently, these mechanisms form a self-reinforcing cycle in which inflammation, metabolic stress, and impaired regeneration perpetuate progressive muscle decline [217].

Although a substantial body of evidence supports these mechanisms, important inconsistencies remain. For example, while many studies report upregulation of proteolytic pathways in COPD muscle, the extent to which autophagy is maladaptive versus compensatory remains debated [218,219]. Similarly, mitochondrial dysfunction has been consistently observed, yet its causal versus secondary role in muscle wasting is not fully resolved [220]. Variability in patient populations, disease severity, nutritional status, and methodological approaches are likely to contribute to these discrepancies. Addressing these inconsistencies requires well-characterized cohorts and standardized outcome measures [221].

From a clinical perspective, these mechanistic insights have important implications. First, they highlight that single-modality interventions are unlikely to be sufficient. Nutritional support alone may partially restore anabolic signaling but will have limited impact in the presence of ongoing inflammation and physical inactivity [132,222]. Conversely, exercise-based rehabilitation improves mitochondrial function and muscle performance but may be blunted by inadequate nutritional intake. This supports a multimodal therapeutic approach integrating targeted nutrition, structured exercise, and optimization of underlying disease control [223,224]. Importantly, these mechanistic and therapeutic responses are not uniform across COPD populations. As detailed in Section 4.3, disease severity (GOLD stage), exacerbation frequency, age, and comorbidities substantially modify both sarcopenia risk and responsiveness to nutritional and rehabilitation interventions [225]. This heterogeneity highlights the limitations of a “one-size-fits-all” approach and supports the need for phenotype-stratified, personalized management strategies incorporating clinical, functional, and metabolic profiling [34]. Second, emerging biomarkers, including circulating microRNAs and proteomic signatures, offer potential for earlier detection and risk stratification, although their clinical utility remains to be validated in large, prospective studies [226].

Despite these advances, several limitations of the current evidence base must be acknowledged. Much of the mechanistic data is derived from small, cross-sectional studies or extrapolated from aging or non-COPD populations, limiting disease-specific interpretation [227]. Interventional trials targeting muscle dysfunction are often short in duration, heterogeneous in design, and underpowered to detect clinically meaningful outcomes. Furthermore, variability in the definitions of sarcopenia and frailty complicates comparisons across studies [228]. As a narrative review, the present work is also subject to potential selection bias and does not provide quantitative synthesis of effect sizes.

Future research should prioritize longitudinal and mechanistically integrated studies that simultaneously assess inflammatory, metabolic, and functional domains. There is a particular need for large, well-designed randomized controlled trials evaluating combined nutritional and exercise interventions, stratified by baseline nutritional and inflammatory status. Advances in multi-omics technologies offer promising opportunities to identify molecular endotypes and enable precision medicine approaches; however, these strategies must be translated into clinically accessible tools. Finally, greater emphasis should be placed on early identification of at-risk individuals, as interventions are likely to be most effective before the establishment of advanced frailty [229].

Taken together, COPD-related sarcopenia and frailty arise from a complex and interconnected network of pathophysiological processes rather than isolated mechanisms. Central to this network is the interaction between systemic inflammation and nutritional impairment, which drives mitochondrial dysfunction, anabolic resistance, and impaired muscle regeneration, ultimately leading to progressive skeletal muscle decline and functional impairment. Recognizing these interdependencies is essential for developing effective, multimodal therapeutic strategies and for improving clinical outcomes in this vulnerable population.

## 7. Conclusions

Sarcopenia and frailty in COPD arise from interacting inflammatory, metabolic, mitochondrial, and lifestyle-related mechanisms, with nutritional impairment playing a key role by promoting proteolysis, suppressing anabolism, and accelerating functional decline. Integrating nutritional assessment and targeted dietary support is therefore clinically important.

Evidence for specific nutritional interventions remains limited, as most trials are small, short-term, and heterogeneous, with few demonstrating sustained benefits on major clinical outcomes. Single-nutrient approaches, particularly antioxidants, have shown minimal effect, supporting the need for multicomponent strategies.

Current data suggest that improving protein and energy intake, correcting deficiencies (especially vitamin D), and using leucine-enriched or multicomponent supplements particularly alongside exercise can enhance muscle mass and function, mainly in nutritionally depleted patients. Future research should prioritize large, well-designed trials, precision nutrition approaches, biomarker validation, and long-term outcomes. Until then, management should remain individualized and multidisciplinary.

## Figures and Tables

**Table 1:** Evidence Linking Systemic Inflammation to Sarcopenia and Frailty in COPD.

Author, Year	Design	Population	COPD Severity	Mechanism	Comparator	Outcomes	Key Findings	Stats	Limitations
Spruit et al., 2003 [48]	Prospective longitudinal + cross-sectional	*n* = 57 (34 hospitalized, 13 stable, 10 controls)	Exacerbated and stable COPD	Systemic inflammation (CXCL8, IL-6, CRP) and IGF-I vs. muscle strength	Stable COPD; healthy controls	QPT, handgrip, PImax, FEV_1_, TLCO, cytokines, IGF-I	Hospitalized patients had lowest strength; QPT declined during admission and partially recovered at 90 days; inflammation inversely and IGF-I positively associated with strength	QPT vs. CXCL8: r = −0.53; QPT vs. IGF-I: r = 0.41; model explained 59% variance (*p* = 0.01)	Observational; steroid use/inactivity not controlled; effort-dependent measures; male-only; attrition risk
Plant et al., 2010 [49]	Cross-sectional biopsy	COPD with severe quadriceps weakness vs. controls (n not clear)	Stable severe COPD	Ubiquitin–proteasome activation (atrogin-1, Nedd4, MuRF1)	Healthy controls	Muscle mRNA/protein expression	↑ Atrogin-1 and Nedd4; MuRF1 unchanged → selective proteolysis activation	Significant differences between-groups	Cross-sectional; selective severe cases; single muscle; unclear sample size
Barreiro et al., 2011 [50]	Cross-sectional	*n* = 48 (COPD + controls)	Moderate–severe COPD	Apoptosis and inflammation in respiratory/peripheral muscle	Sedentary controls	Inflammatory cells, TUNEL, caspase-3, QMVC	↑ Apoptosis in diaphragm (moderate & severe) and quadriceps (severe); inversely related to strength; minimal inflammation	TUNEL ↑ (*p* < 0.05– < 0.001); ANOVA + correlations	Cross-sectional; normal body composition only; small diaphragm sample; TUNEL specificity limits

Abbreviations: CRP: C-reactive protein; CXCL8: chemokine (C-X-C motif) ligand 8 (interleukin-8); FEV_1_: forced expiratory volume in 1 second; IGF-I: insulin-like growth factor I; IL-6: interleukin-6; mRNA: messenger ribonucleic acid; MuRF1: muscle-specific RING finger protein 1; Nedd4: neural precursor cell expressed developmentally downregulated protein 4; PImax: maximal inspiratory mouth pressure; QPT: quadriceps peak torque; QMVC: quadriceps maximal voluntary contraction; TLCO: carbon monoxide transfer factor; TUNEL: terminal deoxynucleotidyl transferase dUTP nick end labelling; ↑ increased; → indicates selective proteolysis activation.

**Table 2:** Evidence on Anabolic–Catabolic Signaling Pathways in COPD-Related Sarcopenia.

**Author, Year**	**Design**	**Population**	**COPD Severity**	**Mechanism**	**Comparator**	**Outcomes**	**Key Findings**	**Stats**	**Limitations**
Barreiro et al., 2005 [61]	Case–control	*n* = 19 (COPD + controls)	Moderate severe (FEV_1_ 62% vs. 42%)	Oxidative stress in diaphragm (carbonyls, HNE, NOS)	Controls; moderate COPD	Oxidative markers; antioxidants; NOS; fiber type; MIP; VO_2_max	Severe COPD showed ↑ carbonyls/HNE; inversely related to FEV_1_ and exercise capacity; ↓ eNOS; ↑ type I fibers linked to oxidation	Carbonyls vs. FEV_1_: r = −0.752; eNOS vs. MIP: r = 0.607 (*p* < 0.05)	Small sample; male-only; surgical biopsies; cross-sectional
Remels et al., 2014 [62]	Translational (human + in vitro)	*n* = 81 (59 COPD)	Moderate–severe (FEV_1_~47%)	NF-κB–mediated ↓ IKK-α → impaired PGC-1/PPAR signaling	Controls; low TNF-α COPD	IKK-α; oxidative enzymes; PGC-1α/PPAR; TNF-α; FEV_1_	↓ IKK-α in COPD (worse with high TNF-α); linked to impaired oxidative phenotype; TNF-α inversely related to muscle function	IKK-α vs. FEV_1_: r = 0.473; TNF-α vs. FEV_1_: r = −0.417	Cross-sectional human data; small subgroups; in vitro limits translation
Vogiatzis et al., 2005 [63]	RCT	*n* = 19	Advanced (FEV_1_~40%)	Exercise-induced muscle remodeling	IE vs. CLE training	Fiber CSA; capillarity; oxidative enzymes; Wpeak; symptoms	Training ↑ fiber CSA, capillarity, and performance; IE ≈ CLE physiologically but ↓ dyspnea	CSA: *p* ≤ 0.008; Wpeak +19% (*p* = 0.04)	Small sample; short duration; no sedentary control

Abbreviations: FEV_1_: forced expiratory volume in 1 second; HNE: 4-hydroxynonenal; NOS: nitric oxide synthase; eNOS: endothelial nitric oxide synthase; MIP: maximal inspiratory pressure; VO_2_max: maximal oxygen uptake; IKK-α: IκB kinase alpha; NF-κB: nuclear factor kappa B; PGC-1α: peroxisome proliferator-activated receptor gamma coactivator 1-alpha; PPAR: peroxisome proliferator-activated receptors; TNF-α: tumor necrosis factor alpha; IE: interval exercise; CLE: continuous low-intensity exercise; CSA: muscle fiber cross-sectional area; Wpeak: peak work rate; RCT: randomized controlled trial. Arrows indicate direction of change: ↑ increase, ↓ decrease, and → association or mechanistic linkage.

**Table 4:** Evidence on hypoxemia and muscle dysfunction in COPD.

Author, Year	Design	Population	COPD Severity	Mechanism	Comparator	Outcomes	Key Findings	Stats	Limitations
Gosker et al., 2002 [82]	Cross-sectional biopsy	*n* = 30 (15 COPD, 15 controls)	Moderate–severe (FEV_1_ < 70%)	Fiber-type redistribution and metabolic profile (COX, SDH, GlyP)	Controls; emphysema vs. non-emphysema	Fiber types; oxidative (COX, SDH) and glycolytic (GlyP) enzymes	↓ Type I fibers and ↑ hybrid fibers in COPD → active transition; ↓ oxidative capacity (incl. within IIa fibers); abnormalities more pronounced in emphysema	Type I: 16% vs. 42%; hybrid: 29% vs. 16% (*p* < 0.05); oxidative capacity correlated with type I (NR)	Small sample; cross-sectional; steroid use (7/15); single center; mechanisms not causal

Abbreviations: FEV_1_: forced expiratory volume in 1 second; COX: cytochrome c oxidase; SDH: succinate dehydrogenase; GlyP: glycogen phosphorylase; IIa: type IIa muscle fibers; NR: not reported. Arrows indicate direction of change: ↑ increased, ↓ decreased, and → indicates transition or association.

**Table 5:** Evidence on glucocorticoid-induced muscle atrophy in COPD.

Author, Year	Design	Population	COPD Severity	Mechanism	Comparator	Outcomes	Key Findings	Stats	Limitations
van Krimpen et al., 2026 [85]	Narrative review	Literature synthesis (studies up to *n* = 5054)	Stable COPD and ECOPD	GC-induced muscle weakness (↓ IGF-1/Akt/mTOR; ↑ FOXO, myostatin, UPS/autophagy)	GC vs. non-GC; stable vs. ECOPD; controls	FFM; strength; ASMI; fiber size; protein turnover markers	GC exposure promotes muscle wasting via anabolic suppression and catabolic activation; associated with ↓ strength and ASMI; pharmacologic targets identified but untested in ECOPD	Handgrip: −0.07 kg/GC (−0.08 COPD); ASMI: −0.01 kg/m^2^; follow-up: −0.18 kg; dexamethasone: −24–33% fiber force; nandrolone RCT: ↑ FFM/strength	Narrative design; heterogeneous studies; no ECOPD-specific trials; no direct biopsy confirmation of GR activation

Abbreviations: ECOPD: exacerbation of chronic obstructive pulmonary disease; GC: glucocorticoids; IGF-1: insulin-like growth factor 1; Akt: protein kinase B; mTOR: mechanistic target of rapamycin; FOXO: forkhead box O transcription factors; UPS: ubiquitin–proteasome system; FFM: fat-free mass; ASMI: appendicular skeletal muscle index; RCT: randomized controlled trial; GR, glucocorticoid receptor; ↑ increased, ↓ decreased.

**Table 6:** Evidence on nutritional deficiency and energy imbalance in COPD.

Author, Year	Design	Population	Intervention	Dose	Duration	Comparator	Outcomes	Key Findings	Stats	Limitations
Machado et al., 2023 [87]	Cross-sectional (CO-SYCONET)	*n* = 2137 COPD	Observational (BIA-based body composition)	NA	NA	BMI-stratified groups	6MWD; HRQL; inflammation; FFMI	Low FFMI common across BMI; FFM linked to 6MWD (underweight); fat mass linked to inflammation; HRQL not independently associated	FFM vs. 6MWD: *p* < 0.05; fat mass vs. inflammation: *p* < 0.05; HRQL: NS	Cross-sectional; BIA misclassification risk; no longitudinal data
Kaluźniak-Szymanowska et al., 2024 [88]	Cross-sectional	*n* = 124 older COPD	Observational (phenotype classification)	NA	NA	Phenotype groups; GOLD stages	6MWT; strength; GOLD distribution	Sarcopenia more frequent in severe COPD; sarcopenia/sarcopenic obesity ↓ 6MWT; supports body composition assessment beyond BMI	Sarcopenia vs. severity: *p* = 0.043; 6MWT differences: *p* ≤ 0.041	Single center; older/male-biased sample; cross-sectional

Abbreviations: CO-SYCONET: COPD and Systemic Consequences cohort network; BIA: bioelectrical impedance analysis; BMI: body mass index; FFMI: fat-free mass index; FFM: fat-free mass; HRQL: health-related quality of life; 6MWD: six-minute walking distance; 6MWT: six-minute walk test; NS: not significant; GOLD: Global Initiative for Chronic Obstructive Lung Disease classification; NA, not applicable/not available; Arrows indicate direction of change: ↓ decreased.

**Table 7:** Evidence on reduced physical activity and rehabilitation in COPD.

Author, Year	Design	Population	Intervention	Dose	Duration	Comparator	Outcomes	Key Findings	Stats	Limitations
Gosker et al., 2002 [82]	Cross-sectional biopsy	*n* = 30 (15 COPD, 15 controls)	Observational	NA	NA	Controls; emphysema vs. non-emphysema	Fiber types; COX/SDH; GlyP; lung function	↓ Type I and ↑ hybrid fibers in COPD → active transition; ↓ oxidative capacity (incl. IIa fibers); more pronounced in emphysema; GlyP higher without steroids	Type I: 16% vs. 42% (*p* < 0.001); hybrid: 29% vs. 16% (*p* = 0.008); COX/SDH ↓ (*p* < 0.001); correlations r = 0.60–0.84	Small sample; cross-sectional; steroid confounding; single center; no activity data
Mador et al., 2004 [149]	RCT (parallel)	*n* = 24 COPD	Combined endurance + strength vs. endurance alone	Progressive loading (combined)	NR	Endurance training	Strength; 6MWD; endurance time; QoL; fatigability	Combined training ↑ muscle strength; endurance alone no strength gain; functional and QoL improvements similar between groups	Strength (quadriceps, lat dorsi): *p* < 0.05 between groups; 6MWD/QoL: within-group *p* < 0.05, between-group NS	Small sample; limited power; no blinding; protocol details NR; no added functional benefit from strength

Abbreviations: COX: cytochrome c oxidase; SDH: succinate dehydrogenase; GlyP: glycogen phosphorylase; RCT: randomized controlled trial; NR: not reported; 6MWD: six-minute walking distance; QoL: quality of life; IIa: type IIa muscle fibers; NS: not significant. Arrows indicate direction of change: ↑ increase, ↓ decrease, and → transition or association.

**Table 8:** Evidence on satellite cell (SC) dysfunction and sarcopenia in COPD and aging.

Author, Year	Design	Population	Intervention	Dose	Duration	Comparator	Outcomes	Key Findings	Stats	Limitations
Vogiatzis et al., 2007 [155]	Prospective pre–post	*n* = 15 COPD	High-intensity endurance training	3 sessions/week	10 w	Pre vs. post	Inflammation (TNF-α, IL-6, CRP); IGF-I, MGF, MyoD; fiber CSA; work rate	↑ Work rate and fiber CSA; ↑ IGF-I/MGF/MyoD (anabolic signaling); no change in inflammatory markers	Work rate +10% (*p* = 0.004); CSA ↑ (*p* = 0.001); IGF-I/MGF/MyoD ↑ (*p* ≤ 0.044); inflammation: NS	Small sample; no control group; non-randomized; training intensity details limited
Thériault et al., 2012 [158]	Cross-sectional biopsy	*n* = 23 (16 COPD, 7 controls)	Observational	NA	NA	Controls; COPD subgroups	Satellite cells; central nuclei; telomere length; MTCSA	↑ Regenerative markers in preserved muscle; ↓ telomere length in COPD (more in atrophy); telomere length correlates with muscle mass	Central nuclei: *p* < 0.001; telomere ↓: *p* < 0.05–0.005; MTCSA correlations: R = 0.435–0.523	Small sample; cross-sectional; advanced COPD only; limited senescence markers; confounders not controlled

Abbreviations: TNF-α: tumor necrosis factor alpha; IL-6: interleukin-6; CRP: C-reactive protein; IGF-I: insulin-like growth factor I; MGF: mechano growth factor (IGF-IEc); MyoD: myogenic differentiation factor 1; CSA: cross-sectional area; MTCSA: muscle fiber cross-sectional area; NS: not significant. Arrows indicate direction of change: ↑ increased, ↓ decreased.

**Table 9:** Evidence on vascular impairments in aging skeletal muscle and COPD.

Author, Year	Design	Population	Intervention	Dose	Duration	Comparator	Outcomes	Key Findings	Stats	Limitations
Eskurza et al., 2004 [164]	Randomized crossover	Young and older men (sedentary and trained)	IV and oral ascorbic acid	IV + oral dosing	Acute; 30 d	Young; trained; pre–post	FMD; NTG; oxidative markers	Aging ↓ endothelial function via oxidative stress; IV vitamin C restores FMD; training preserves function; oral supplementation ineffective	FMD ↓ in older vs. young (*p* < 0.01); restored with IV (NS vs. young)	Healthy males only; conduit artery; not COPD
Casey & Joyner, 2012 [24]	Narrative/mechanistic review	Healthy humans (multiple studies)	Pharmacologic modulation	Various	Acute	Young; normoxia	Blood flow; vasodilation; NO signaling	Compensatory vasodilation during hypoxia is NO-mediated; not primarily driven by sympatholysis or adenosine	~20% flow ↑ per ~20% ↓ O_2_ sat; *p* < 0.05 in key studies	Not COPD; small-muscle model; indirect translation
Iepsen et al., 2017 [173]	Case–control (invasive)	COPD vs. healthy controls	Observational ± SNP/ACh testing	SNP/ACh infusion	Acute	Healthy controls	Leg blood flow; O_2_ delivery; endothelial markers	COPD shows ↓ exercise muscle blood flow and O_2_ delivery; dysfunction linked to ↑ endothelin-1 and ↓ prostacyclin, not impaired vasodilatory capacity	Blood flow and O_2_ delivery ↓ (*p* < 0.05); endothelin-1 ↑ (*p* < 0.05); prostacyclin response absent	Small sample; invasive; cross-sectional; limited workload

Abbreviations: COPD: chronic obstructive pulmonary disease; IV: intravenous; FMD: flow-mediated dilation; NTG: nitroglycerin; NO: nitric oxide; SNP: sodium nitroprusside; ACh: acetylcholine; O_2_: oxygen; Arrows indicate direction of change: ↑ increased, ↓ decreased.

**Table 10:** Phenotype-guided nutritional and rehabilitation strategies for sarcopenia and frailty in COPD.

Phenotype	Key Risk Profile	Nutritional Strategy	Rehabilitation Strategy
Mild–moderate COPD (GOLD 1–2), stable	Early inactivity, mild anabolic resistance	Protein ≥ 1.2 g/kg/day; leucine-rich diet; vitamin D optimization	Early activity promotion; structured exercise initiation
Severe COPD (GOLD 3–4), stable	Hypoxemia, inflammation, low FFMI	Protein 1.2–1.5 g/kg/day; energy 35–45 kcal/kg/day; ONS if weight loss/BMI < 21	Combined aerobic + resistance PR; post-exercise protein timing
Frequent exacerbators	Recurrent catabolic episodes, steroid exposure	Peri-exacerbation supplementation; HMB/leucine-enriched ONS	Early post-exacerbation PR (≤4 weeks); functional recovery focus
Older adults (≥70 years), frail	Inflammaging, anabolic resistance, multimorbidity	Protein ≥ 1.5 g/kg/day; ≥0.4 g/kg/meal; vitamin D ± calcium	Multicomponent PR (resistance + balance + endurance); falls prevention
COPD + CKD (stage 3–4)	Renal catabolism, protein restriction limits	Nephrology-guided protein (0.6–0.8 g/kg/day CKD 3–4)	Renal-adapted exercise; moderate progression
COPD + T2DM	Insulin resistance, anabolic impairment	Low-GI ONS; protein distribution with meals	Resistance training prioritized; metabolic conditioning
COPD + Osteosarcopenia	Bone loss, corticosteroid effects, fracture risk	Protein + vitamin D (800–2000 IU) + calcium (1000–1200 mg)	Balance + resistance training; falls prevention

Abbreviations: GOLD: Global Initiative for Chronic Obstructive Lung Disease; FFMI: fat-free mass index; ONS: oral nutritional supplements; BMI: body mass index; PR: pulmonary rehabilitation; HMB: β-hydroxy β-methylbutyrate; CKD: chronic kidney disease; T2DM: type 2 diabetes mellitus; GI: glycemic index; IU: international units.

**Table 11:** Evidence on novel biomarkers of sarcopenia and frailty in COPD.

Author, Year	Design	Population	Intervention	Dose	Duration	Comparator	Outcomes	Key Findings	Stats	Limitations
Sepúlveda-Loyola et al., 2021 [199]	Cross-sectional	COPD and matched controls (older adults)	Observational	NA	NA	Healthy controls	Oxidative/antioxidant markers; muscle mass/strength; function	↓ Antioxidant capacity linked to ↓ muscle mass/strength; oxidative stress higher in COPD; low TRAP/AOPP predict sarcopenia	r = 0.50–0.64 (*p* < 0.05); TRAP OR 8.3; AOPP OR 14.0	Small sample; cross-sectional; multiple comparisons; confounding not fully controlled
Deng et al., 2022 [195]	Observational cohort	Stable COPD	Observational	NA	NA	Internal comparison	GDF-15; muscle mass; strength; ultrasound; sarcopenia	↑ GDF-15 associated with ↓ muscle mass/strength; good diagnostic accuracy; model improves prediction	r = −0.20 to −0.34 (*p* ≤ 0.031); AUC 0.80–0.83; model > 0.89	No controls; cross-sectional; confounding (comorbidities); criteria variability
Karim et al., 2021 [200]	Prospective longitudinal	Male COPD and controls (older adults)	Pulmonary rehabilitation	60–80% peak work rate	6 mo	Healthy controls	CAF22; BDNF; GDNF; function; sarcopenia	COPD: ↑ CAF22, ↓ BDNF/GDNF; PR partially reverses; biomarker panel improves diagnosis	Biomarkers *p* < 0.05; AUC 0.75–0.81; panel 0.81	Male-only; BIA limitations; confounders; no PR control group

Abbreviations: TRAP: total radical-trapping antioxidant parameter; AOPP: advanced oxidation protein products; OR: odds ratio; GDF-15: growth differentiation factor 15; AUC: area under the receiver operating characteristic curve; CAF22: C-terminal agrin fragment 22; BDNF: brain-derived neurotrophic factor; GDNF: glial cell line-derived neurotrophic factor; PR: pulmonary rehabilitation; BIA: bioelectrical impedance analysis; NA: not applicable. Arrows indicate direction of change: ↑ increased, ↓ decreased.

**Table 12:** Evidence on blood biomarkers linked to muscle dysfunction in COPD.

Author, Year	Design	Population	Intervention	Dose	Duration	Comparator	Outcomes	Key Findings	Stats	Limitations
Gao et al., 2022 [206]	Observational (development/validation)	Stable COPD	Observational	NA	NA	Internal comparison	Resistin; TNF-α; muscle mass; strength; ultrasound; sarcopenia	↑ Resistin associated with ↓ muscle mass/strength; predicts sarcopenia better than TNF-α; pro-inflammatory mechanism (NF-κB-related)	r = 0.25 (*p* = 0.007); AUC 0.82–0.83; TNF-α AUC 0.62	No controls; cross-sectional; confounding (comorbidities); criteria variability
Sepúlveda-Loyola et al., 2021 [199]	Cross-sectional	COPD and controls (older adults)	Observational	NA	NA	Healthy controls	Oxidative markers; muscle mass/strength; function	↓ Antioxidant capacity linked to ↓ muscle mass/strength; oxidative stress ↑ in COPD; predicts sarcopenia	r = 0.50–0.64 (*p* < 0.05); OR 8.3–14.0	Small sample; cross-sectional; multiple comparisons; confounding
Winter et al., 2021 [207]	Case–control	COPD, asthma, and controls	Observational	NA	NA	Controls; asthma subgroups	Hemopexin; acute-phase proteins; inflammation	Hemopexin strongly discriminates COPD vs. controls/asthma; reflects systemic inflammation (not muscle-specific)	AUC 0.97–0.98; others *p* < 0.05	No muscle outcomes; cross-sectional; limited mechanistic link to sarcopenia

Abbreviations: TNF-α: tumor necrosis factor alpha; NF-κB: nuclear factor kappa B; AUC: area under the receiver operating characteristic curve; OR: odds ratio. Arrows indicate direction of change: ↑ increased, ↓ decreased.

**Table 13:** Body composition parameters in COPD patients (*n* = 467) before pulmonary rehabilitation program.

Parameter	Mean ± SD
Height (cm)	169.5 ± 12.9
Weight (kg)	80.8 ± 21.8
BMI (kg/m^2^)	27.2 ± 6.5
Body fat (%)	35.2 ± 10.9
Skeletal muscle (%)	26.4 ± 5.3
Resting metabolic rate (kcal)	1615.8 ± 312.2
Visceral fat (index)	11.4 ± 5.3

Abbreviations: BMI, body mass index; SD, standard deviation. Values are expressed as mean ± standard deviation (SD). Data represent COPD patients who participated in the pulmonary rehabilitation program.

## Data Availability

No new data were created or analyzed in this study.

## Abbreviations

Akt	Protein kinase B
AMPK	AMP-activated protein kinase
ASM	Appendicular skeletal muscle mass
ATP	Adenosine triphosphate
BMI	Body mass index
BCAA	Branched-chain amino acids
CAF22	C-terminal agrin fragment-22
CASP	Critical Appraisal Skills Programme
CK	Creatine kinase
COPD	Chronic obstructive pulmonary disease
CRP	C-reactive protein
CT	Computed tomography
DHEA	Dehydroepiandrosterone
Dkk-3	Dickkopf-related protein 3
EWGSOP	European Working Group on Sarcopenia in Older People
FI	Frailty Index
FOXO	Forkhead box O transcription factor
FGF	Fibroblast growth factor
HIF	Hypoxia-inducible factor
IGF-1	Insulin-like growth factor-1
IL	Interleukin
mTOR	Mechanistic target of rapamycin
mTORC1	mTOR complex 1
MRI	Magnetic resonance imaging
MuRF-1	Muscle RING finger-1
MSTN	Myostatin
NF-κB	Nuclear factor kappa-B
NO	Nitric oxide
NOS	Newcastle–Ottawa Scale
PaO_2_	Arterial oxygen tension
PI3K	Phosphoinositide 3-kinase
PRISMA	Preferred Reporting Items for Systematic Reviews and Meta-Analyses
RNS	Reactive nitrogen species
ROS	Reactive oxygen species
SC	Satellite cell
SD	Standard deviation
TNF-α	Tumor necrosis factor-alpha
UPS	Ubiquitin–proteasome system
